# Isolated hypoplastic circumflex coronary artery: a rare cause of haemorrhagic myocardial infarction in a young athlete

**DOI:** 10.1186/1746-1596-8-91

**Published:** 2013-06-06

**Authors:** Florian-Nikolaus Riede, Stefan Bulla, Sebastian Grundmann, Martin Werner, Urs-Nikolaus Riede, Claudia Otto

**Affiliations:** 1 Department of Cardiology, University Hospital Basel, Petersgraben 3, CH-4031 Basel, Switzerland; 2 Department of Diagnostic Radiology, University Hospital Freiburg, i. Br., Hugstetterstr. 55, D-79106 Freiburg i. Br., Germany; 3 Department of Internal Medicine, University Hospital Freiburg, i. Br., Hugstetterstr. 55, D-79106 Freiburg i. Br., Germany; 4 Department of Pathology, University Hospital Freiburg, i. Br., Breisacherstr. 115a, D-79106 Freiburg i. Br., Germany

**Keywords:** Isolated hypoplasia of the left circumflex artery, Myocardial haemorrhagic infarction, Sudden death in young athletes, Coronary artery development

## Abstract

Hypoplastic coronary artery disease is a rare condition that may lead to myocardial infarction and sudden death. Here we describe for the first time an isolated hypoplasia of the left circumflex artery (LCX). An otherwise healthy and athletically active 16-year-old boy was admitted to the intensive care unit (ICU) after out-of-hospital cardiac arrest. He died 12 hours after the initial event. Autopsy revealed an isolated hypoplastic LCX and acute haemorrhagic infarction in the posterolateral myocardium. The existence of isolated hypoplasia of the LCX challenges our understanding of coronary artery development.

**Virtual slides:**

The virtual slide(s) for this article can be found here: http://www.diagnosticpathology.diagnomx.eu/vs/1558483061962648

## Background

Congenital coronary artery anomalies are rare, affecting approximately 1% of the general population [1-6]. They are often an incidental finding in asymptomatic patients [1,5-8]. They occur in 1% of all congenital heart disease and manifest a wide variety of disorders [9-23]. Some, such as the anomalous location of a coronary ostium [22,24] the duplication of coronary arteries, a single coronary artery [18] or multiple coronary ostia [25], become clinically significant only when another surgical cardiac procedure becomes necessary; surgical correction is not generally required in these patients. Approximately 20% of coronary anomalies can lead to life-threatening complications, including myocardial infarction, arrhythmia, or sudden death early in life or during adulthood [21,22,26,27]. On the other hand, the diagnosis of an anomalous origin of the left coronary artery from the pulmonary artery or from the opposite sinus with an inter-arterial course is an indication for surgery. Some anomalies like coronary artery fistulas, myocardial bridging, and coronary aneurysm require surgery only when they cause clinical symptoms [28,29].

Hypoplastic coronary artery disease (HCAD) was first reported in 1970. It occurs rarely and refers to the underdevelopment of one or both coronary arteries or their main branches [20,22,27,30-32]. Hypoplasia of the left circumflex coronary artery is a genuine rarity [7,33] On the basis of angiography, coronary artery anomalies are classified in seven patterns [19,20]. Most of the affected patients were young adults and experienced sudden cardiac death without antecedent symptoms [8,34]. Diagnosis is often made at autopsy [8,24,26,35].

We hereby present the case of sudden death in a 16-year-old healthy Caucasian male who was physically active and participated in sports including competitive mountain-biking. There was no remarkable medical history. This is the first description of an isolated hypoplasia of a coronary artery branch with no other abnormalities of the other coronary arteries or concomitant congenital heart disease.

## Case presentation

A 16-year-old male was admitted to the intensive care unit of our university hospital after out-of-hospital cardiac arrest. While riding his bicycle through the city center, he suddenly collapsed and when he failed to regain consciousness, cardiopulmonary resuscitation was immediately initiated by bystanders and sustained until the arrival of a medical response team 10 minutes later. The initially-detected heart rhythm revealed ventricular fibrillation and cardiopulmonary resuscitation was continued according to current guidelines including intubation, a total of eleven biphasic shocks and a fractionated total dose of 600 mg amiodarone until a stable sinus rhythm could be maintained. However, the patient remained pulse-less, so systemic fibrinolysis was initiated by infusing 8000 units of tenecteplase. Therapeutic hypothermia was induced by the infusion of cold saline solution and he was transported under continuous ventilation and chest compression to our hospital’s ICU, where he arrived 56 minutes after the initial event.

Upon admission, emergency echocardiography ruled out pericardial effusion, however, it showed a severely reduced left ventricular ejection fraction as the reason for the persistent electromechanical dissociation. Because of the known positive predictors for a promising neurological outcome (immediate initiation of bystander-CPR and young age), a pump-driven extracorporal membrane oxygenator was implanted by cannulating the femoral vein and artery during continuous CPR, and he eventually became haemodynamically and respiratorily stable.

**Figure 1:**
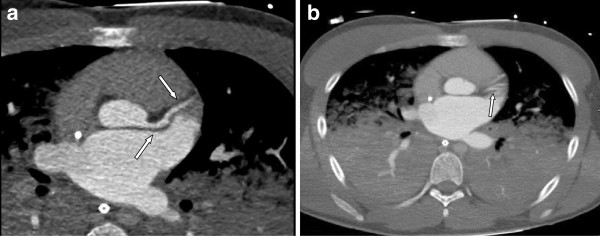
**Segment V, VI (a) and IX (b) are shown by computed tomography.** The outlet of the LCX is not presentable. Lower lobes are atelectatic and signs of pulmonary edema are visible.

Clinical examination of the now stable patient was unremarkable other than the right pupil’s being dilated and unresponsive to light. Due to this finding and his low cardiovascular risk profile, coronary angiography was postponed to perform a head and body high resolution contrast CT, including visualisation of the coronary anatomy. The main findings of the cardio-CT were a rarefication of the left coronary circumflex with normal anatomy of the left anterior descending (LAD) and a prominent right coronary artery (Figure 1). Unfortunately, computed tomography of the head (CCT) imaging also displayed already extensive, diffuse, cerebral oedema with herniation and brain stem compression. Cerebrovascular Doppler-imaging revealed little residual cerebral perfusion. Shortly thereafter, the patient suffered an extensive pulmonary haemorrhage, and his blood pressure could not be stabilized. Once extracorporal circulation was discontinued, the patient was declared dead twelve hours after the initial event.

Initial laboratory testing showed a normal blood count besides a mild thrombocytopenia. Before the event, the patient was well and had no medical history besides a syncopal fall 3 years earlier. There was no family history of sudden cardiac or unexplained death.

Postmortem examination was performed as described by H. Hamperl [36]. The heart was dissected, yielding tangential and transversal transsections, and preserved in formalin for further examination. Tissue sections were stained with hematoxilin/eosin and Elastica van Gieson, as well as with PAS to stain the microthrombi, and Luxol fast blue to stain the ischaemic lesions [37,38]. The necrotic cardiomyocytes were immunohistochemically stained with a monoclonal antibody to C4d (C4d/TK; dilution m1:10, Biozol, Eching, Germany), the cardiomyocytic cellular membrane (sarkolemma) as well as the Z-band were stained with a monoclonal antibody to CD56 (C56/TK; dilution m1:10, Biozol, Eching, Germany) and the myocardial capillaries were stained with the monoclonal antibody CD34(C56/TK; dilution m1:10, Biozol, Eching, Germany) in an Autostainer 9421 (DAKO) according to the manufacturer’s instruction [39-42]. The coronary diameter was measured by a fixed threshold method from the transverse sections obtained along the long-axis of each coronary artery [12,15] after fixation of the heart with formalin.

**Figure 2:**
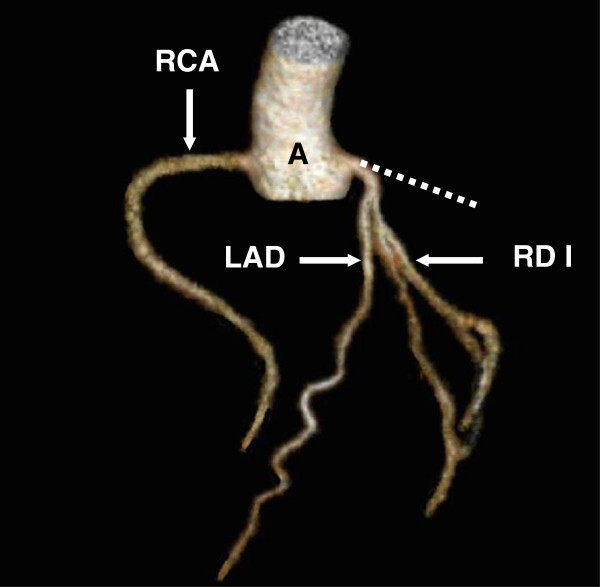
**Volumen rendering technique shows aortic root (A), RCA, LAD and ramus diagonalis I (RD I).** Hypoplastic LCX is not visible, the ordinary position of the LCX is marked as a dotted line.

Initial computed tomography of the head (CCT) and the heart were performed after a pump-driven extracorporal membrane oxygenator was implanted. First, spiral CT of the head was performed without application of contrast agent, to detect a potential intracranial haemorrhage, edema or ischemia. Afterwards ECG-gated contrast enhanced Cardio-CT was performed in standard-technique [43,44]. To obtain a high opacification of the coronary arteries bolus tracking was applied. This means a repetitive measuring of threshold in an interval of 1 second in a region of interest placed in the ascending aorta (ROI), data acquisition was started 6 s after a threshold of 90 Hounsfield units (HU) was reached. Cardio-CT images were transferred to a workstation to generate images in additional orientations and 3D images (Figure 2).

**Figure 3:**
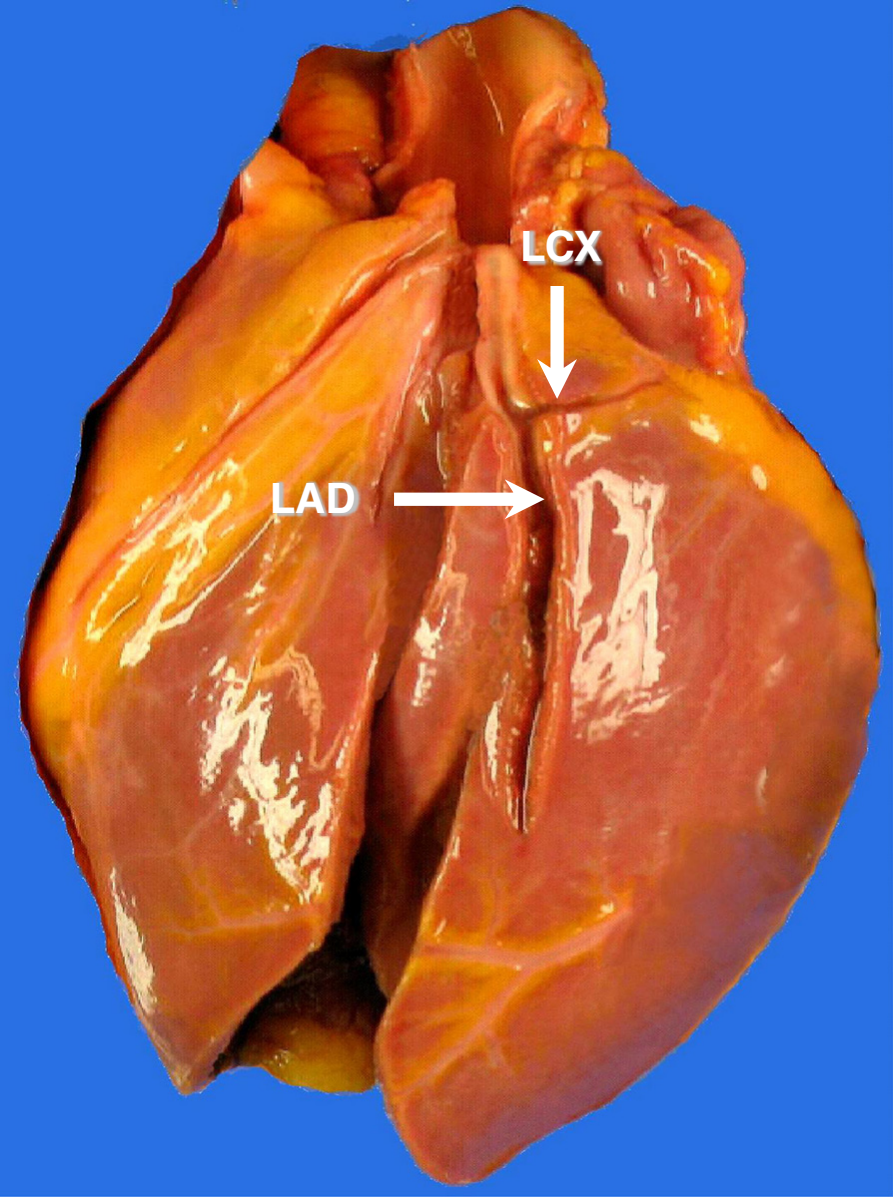
**The heart of a 16-year-old boy.** The LAD is normally developed. The LCX is stenotic and shortened (unfixed heart, antero-lateral view).

At autopsy of the boy’s heart (body weight 87 kg, body length 189 cm), it weighed 280 g. His right coronary had a diameter of 4.5 mm without dominance of the left coronary artery. The left coronary artery originated from the left coronary sinus of the aorta with a single initial trunk. The left coronary artery revealed an average diameter of 4.5 mm and gave rise to the left anterior descending artery with a diameter of 4.3 mm; 5.1 cm behind the left coronary ostium, the LAD provided a single left circumflex coronary artery, showing a diameter of under 1 mm, a reduced length of 2.3 cm and a thin hypoplastic media [4,9] (Figure 3). The LCX provided no origin to different branches [15] along its truncated course. Histologically there were neither signs of previous thrombosis nor vascular inflammation. Preparation of all coronary arteries revealed neither fatty streaks nor sclerosis.

**Figure 4:**
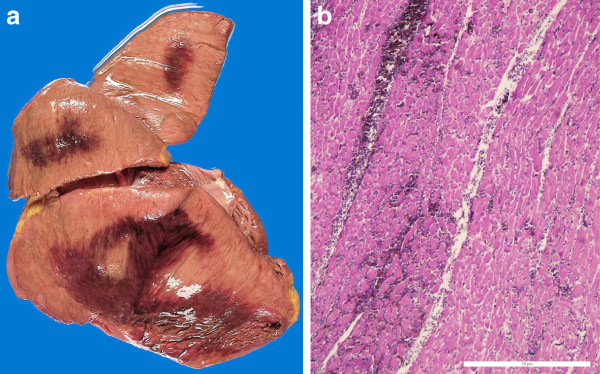
Fresh haemorrhagic areas visible on tangential slices through the posterior wall of the left ventricle: a) macroscopic aspect in the unfixed heart, b) histological aspect in H&E staining (4 ×).

Macroscopically, the heart presented a slightly hypertrophic left ventricle. Areas of haemorrhagic myocardial infarction were visible in the lateral wall (measuring 5 cm × 7 cm) and in the left ventricle’s posterior wall (measuring 4 cm × 3 cm) (Figure 4). Histology revealed cardiomyocytes with contracting band necrosis und necrotic cardiomyocytes without nuclei and obvious cardiomyocytic waving in the border area. This necrotic zone was on the border of vital muscle demarcated by leucocytes. The myocardial capillaries stained with CD34 in the left ventricle’s posterior wall revealed a smaller diffusion distance to the nearest capillary compared with the vital myocardium of the anterior left ventricular wall [15]. Analogously, the cardiomyocytes in the posterolateral wall were much smaller in diameter and located far apart from one another. The expression of CD56 revealed a pericellular pattern. In addition to these cardiac findings, the small and large intestines were distended and totally infarcted.

## Conclusions

Autopsy of this 16-year-old athlete showed a posterolateral myocardial infarction in a focus of ischaemic cardiomyopathy. This infarction was provoked by isolated hypoplasia of the LCX with significant shortening.

In the LCX’s supply area, the cardiomyocytes were reduced in size and disassociated. They revealed a pericellular expression pattern for CD56 typical of ischaemia-related intercellular loosening of the cardiomyocytic bond characteristic of ischaemic cardiomyopathy [39,40]. This led us to conclude that the stenotic LCX hypoplasia must have existed for quite some time, leading to a mildly hypoplastic left ventricle and focal ischaemic cardiomyopathy. As we observed no atherosclerotic lesions in the hypoplastic LCX, we assume that congenital hypoplasia was responsible for the stenosis and shortened LCX. The fact that hypoplastic coronary arteries have both a small luminal diameter (usually <1 mm) and reduced length supports this assumption [14].

The myocardial infarction was haemorrhagic and surrounded by an alterative myocarditis. That means that the infarction of the myocardium was due to an ischaemic period longer than 24 h, leading to cardiogenic shock and to an acute infarction of the intestine and consecutive ileus.

Haemorrhagic myocardial infarctions are rare. Intramyocardial hemorrhage often occurs in conjunction with reperfusion [18,19,24]. Studies on dogs have shown that if reperfusion is instituted less than 20 minutes after a coronary occlusion, myocardial blood flow, function and histology return to normal. However, if 40 minutes or more elapse before blood flow is restored, normal perfusion may not be restored and histology may fail to recover [45,46]. In our case, the haemorrhagic myocardial infarction can be attributed to the 56-minute interval after the initial event in addition to the fibrinolysis [45-47].

The etiology of the HCAD is still unknown [48-52], though it is postulated that it results from various conditions, including stenosis of the coronary artery orifice, an aberrant course between the pulmonary artery and aorta, a coronary artery ostium in ectopic position; there is also the hypothesis of a stenosis of the coronary ostium [22,23,25,32]. Since the boy’s autopsy revealed both a non-stenotic coronary ostia and thin coronary vessel wall with hypoplasia of the media layer, we speculate that an abnormal proliferative response by angiogenetic cells in the coronary artery (i.e. LCX) may be involved in the pathogenesis of this disease [30] rather than coronary artery spasm reflecting abnormal vasodilatory mechanisms and endothelial dysfunction [33,52]. Mice with an endothelial nitric oxid synthase (NOS3) genetic deficit provide a molecular basis with which to explain HCAD. The NOS3 deficiency results in a condition mirroring HCAD in humans [53]. Recent experiments suggest that NOS3 promotes normal coronary artery development via increases in the expression of transcription and growth factors (such as VEGF, bFGF, EPO) and the migration of epicardium-derived cells into the myocardium [53]. Other studies have shown that an increased risk of congenital heart disease, especially conotruncal heart defects, is associated via a reduction in the NOS3 activity induced by a common 894G > T single nucleotide polymorphism [54]. In addition, environmental factors and maternal conditions including psychological stress, diabetes mellitus and hypertension, which decrease NOS3 expression and/or activity, are associated with the increased risk of congenital heart disease [53-55]. These factors together with our findings from this case lead us to this hypothesis as to the etiology of isolated LCX hypoplasia:

1. Myocardial angiogenesis was influenced at a very late stage of heart development, thus the left ventricle and LAD could develop normally, while the LCX became hypoplastic and shortened;

2. Myocardial angiogenesis proceeded in certain morphogenetic fields of the heart at different periods of time.

## Consent

Written informed consent was obtained from the parents of the patient for publication of this Case Report and any accompanying images. A copy of the written consent is available for review by the Editor-in-Chief of this journal.

## Abbreviations

CCT: Computed tomography of the head; CPR: Cardiopulmonary resucitation; HCAD: Hypoplastic coronary artery disease; ICU: Intensive care unit; LAD: Left anterior descending coronary artery; NOS3: Endothelial nitric oxid synthase; LCX: Left circumflex artery.

## Competing interests

There are no financial and non-financial interests to declare in relation to this manuscript.

## Authors’ contributions

FNR participated in the sequence alignment and drafted the manuscript. SB carried out the radiological analysis of the case to be published. SG provided the clinical information of the case to be published. MW carried out the final approval of the version to be published. UNR conceived of the study and participated in its design and coordination. CO carried out the autopsy and the histological analysis of the case presented; she also participated in the drafting of the manuscript. All authors read and approved the final manuscript.
